# Stent Placement in a Neonate with Sano Modification of the Norwood using Semi-Elective Extracorporeal Membrane Oxygenation

**DOI:** 10.5935/abc.20160080

**Published:** 2016-12

**Authors:** Mustafa Gulgun, Michael Slack

**Affiliations:** Associated Profesor - Children National Medical Center - Division of Pediatric Cardiology, Washington, District of Columbia, USA

**Keywords:** Infant, Newborn, Hypoplastic Left Heart Syndrome / surgery, Extracorporeal Membrane Oxygenation, Heart Defects, Congenital / surgery, Stent

## Abstract

Extracorporeal membrane oxygenation (ECMO) is a well-established tool of cardiopulmonary circulatory support for cardiopulmonary failure in children and adults. It has been used as a supportive strategy during interventional procedures in neonates with congenital heart disease. Herein, we describe a neonate with hypoplastic left heart syndrome who underwent stenting of the Sano shunt and left pulmonary artery after Norwood Sano operation using intra-procedural ECMO support. The use of ECMO as a bridge to recovery might be a feasible and reasonably safe adjunctive approach in the treatment of complications in selective case of neonates having undergone the Norwood Sano procedure.

## Introduction

Extracorporeal membrane oxygenation (ECMO) is a mechanical form of cardiopulmonary circulatory support used as bridging therapy for profound cardiopulmonary failure in children and adults. Indications for ECMO have been expanded since its first usage in the early 1970s.^1^ Today, the utility of ECMO as a supportive strategy for high-risk interventional procedures in infants with congenital heart disease is expanding in practice.^2,3^ Herein, we present a neonate with hypoplastic left heart syndrome (HLHS) who underwent stenting of the Sano shunt (SS) and left pulmonary artery (LPA) after Norwood Sano procedure using intra-procedural ECMO support.

## Case Report

**Figure 1:**
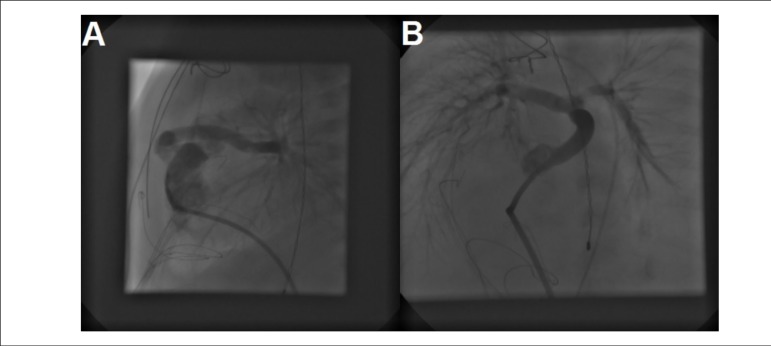
Lateral view of right ventriculogram showing the proximal Sano shunt, the acute angulation and suspected kink in the shunt as it bends behind the sternum (A). Cranial view of the left pulmonary artery showing a severe stenosis, seen as a very pale area in the proximal artery (B).

**Figure 2:**
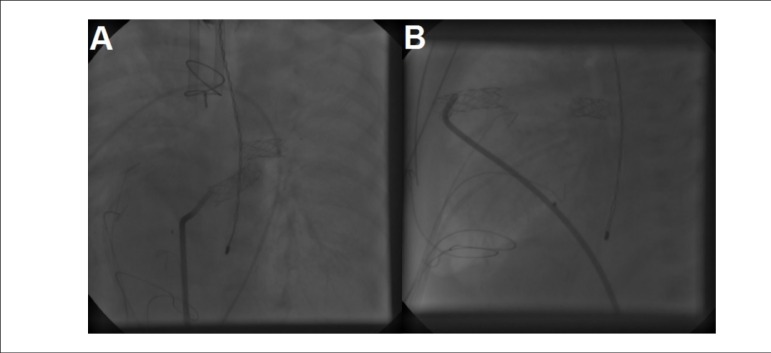
Access the video through the link: http://www.arquivosonline.com.br/2016/english/10706/video_ing.asp Normal caliber and good placement of the Sano shunt, left pulmonary artery stent in the anteroposterior (A) and lateral position (B), and appropriate position of the arterial and venous cannulas.

A newborn male, born with HLHS (mitral and aortic atresia) and mesocardia underwent a Stage 1 Norwood Sano procedure at age 10 days. Due to his cardiac malposition, multiple attempts to close the chest were unsuccessful. Eventually, his chest was partially closed (skin only) 8 days after the operation, and over the subsequent days, there was increasing cyanosis and need for additional inotropic support. On day 16, the neonate was referred to the cardiac catheterization laboratory for the assessment of post-operative anatomy and reasons for progressive hemodynamic compromise and hyper-cyanosis. Informed consent was obtained from his parents for the cardiac catheterization and possible interventions. An angiogram performed with a catheter positioned at the origin of the SS in the right ventricle (RV), demonstrated an acute angle take-off from the RV with a suspected kink in the shunt just behind the sternum. The main body of the shunt was patent. However, there was also a severe LPA stenosis resembling a filling defect due to a local thrombus formation. The right pulmonary artery (RPA) was normal (Figure 1, videos 1 and 2). The diameter of the mid-SS, RPA, and LPA measured 4.6 mm, 4.7 mm and 2.1 mm, respectively. With the neonate under general endotracheal anesthesia and ventilated with 100% oxygen, a 5 x 20 mm Sterling balloon (Boston Scientific, Natick, MA) was advanced over a 0.018 inch wire across the proximal LPA and inflated twice. During two brief balloon inflations, the neonate experienced severe hemodynamic embarrassment and required resuscitation with brief chest compressions and bolus epinephrine after the second inflation. An immediate post balloon angiogram demonstrated no acute vascular complication. However, no improvement in the LPA caliber was seen, which confirmed that the stenosis was not due to thrombus formation. After consultation with the cardiac surgeon (including consideration of the extreme difficulty in getting the chest closed after the Stage 1 palliation), the neonate was placed on temporary venoarterial ECMO on a semi-elective basis to further interventional treatment (Figure 2).

**Video 1:** To view the video click on the link: http://www.arquivosonline.com.br/2016/english/10706/video_ing.asp Right ventricle angiogram in lateral position showing a normal caliber Sano shunt with a sharp, less than 90-degree take-off from the right ventricle.

**Video 2:** To view the video click on the link: http://www.arquivosonline.com.br/2016/english/10706/video_ing.asp The Sano shunt angiogram in antero-posterior position showing a severe left pulmonary artery stenosis.

After successfully deploying ECMO via the right internal jugular and right carotid artery, an initial attempt at stenting the LPA was made. The initial stent (5 mm x 12 mm Palmaz Blue; Cordis Corp., Freemont, CA) was advanced through the SS over a 0.018 guidewire, however, due to the severe angulation between the SS and the LPA origin, the stent slipped off the balloon and was retrieved percutaneously.

A second attempt was made by placing the 0.018 inch wire into the LPA which resulted in successful stent (5 mm x 12 mm Palmaz Blue; Cordis Corp., Freemont, CA) deployment in the LPA. The acute angulation in the proximal SS was corrected using a 6 mm x 18 mm stent (Palmaz Blue; Cordis Corp., Freemont, CA). A follow-up, hand injection angiogram demonstrated appropriate placement of the stents, with normal caliber and good flow in both LPA and proximal SS (Figure 2, video 3).

**Video 3:** To view the video click on the link: http://www.arquivosonline.com.br/2016/english/10706/video_ing.asp Angiogram showing normal caliber and appropriate placement of the Sano stent and left pulmonary artery stent. There is appropriate filling indicating improvement of the left pulmonary artery flow and slight improvement in pulmonary artery arborization in the left lung.

Although the patient was decannulated 4 days after the intervention, the patient had hemodynamic instability requiring escalation of vasopressor support and multiple fluid boluses for a while. At the time of the subsequent bidirectional Glenn operation about 2 months after the stent implantation, the stent was removed and the LPA underwent surgical repair. Subsequently, a stent angioplasty of the LPA was required. The neonate was treated using a stent with a diameter that goes up to adult size with an excellent outcome.

## Discussion

Conventional cardiopulmonary resuscitation as an initial intervention for severe hemodynamic compromise during interventional catheterization procedures is common and often unsuccessful for cardiac arrest in patients with single ventricle physiology. The use of ECMO in addition to the conventional cardiopulmonary resuscitation as a circulatory support in the early postoperative period after Norwood Sano operation can improve survival in neonates.^4^ ECMO as a bridge to recovery can also be useful in selected situations in which surgical interventions are considered to be of excessive risk. Interventional catheter-based treatment may be possible if hemodynamic stability can be enhanced with additional ECMO support and cardiac arrest prevented during the catheter maneuvers.^1-4^ The utilization of ECMO in this setting may provide enough hemodynamic stability and evidence for the surgeons to perform accurate diagnosis and effective therapeutic interventions. Moreover, reports have indicated that elective ECMO prior to cardiac arrest and end-organ damage in neonates with single-ventricle physiology resulted in improved outcomes.^5,6^ In our case, although the coronary stent was not an ideal long term solution for LPA stenosis, the ECMO support was chosen because of patient's clinical situation at the time of the catheterization.

The use of ECMO during the therapeutic cardiac interventions resulted in the successful repair of the proximal SS and after complicated Stage 1 Norwood Sano palliative surgery. This ECMO strategy prevented the recurrence of hemodynamic instability during stenting procedure, and increased the chances of recovery. Overall, in selected situations, the use of ECMO may be a feasible and reasonably safe adjunctive strategy in the treatment of complications in neonates following the Norwood Sano procedure.
